# Single Center-Based Real-World Experience on Anti-IL 1 Biological Response Modifiers: A Case Series and Literature Review

**DOI:** 10.3390/children11091146

**Published:** 2024-09-22

**Authors:** Olcay Y. Jones

**Affiliations:** 1 Walter Reed National Military Medical Center, Bethesda, MD 20889, USA; olcay.y.jones.civ@health.mil; Tel.: +1-301-648-0641; 2 Department Pediatrics, Uniformed Services University of the Health Sciences, Bethesda, MD 20814, USA; 3 Department Rheumatology, George Washington University, Washington, DC 20037, USA

**Keywords:** inflammation, IL-1, anti-IL-1 biologic response modifiers, anakinra, canakinumab

## Abstract

Background: This communication summarizes our single-center experience with the use of anti-IL-1 biologic response modifiers for treating autoimmune and autoinflammatory conditions in children. Methods: We outline our rationale for the off-label use of anakinra and discuss emerging treatment paradigms that necessitate further research and validation. Results: Anakinra has enabled personalized treatment, whether used as a single agent on an as-needed basis, as part of a background treatment regimen, or in combination with colchicine. Our data also highlight the significance of anakinra in treating post-infectious inflammatory diseases, demonstrating its high efficacy in novel applications such as rheumatic fever and post-viral arthritis. Canakinumab, on the other hand, has provided long-term remission. Both medications were well-tolerated, with no serious adverse effects reported. Conclusions: Based on our observations and successful outcomes, we advocate for future collaborative efforts to improve access to anti-IL-1 medications to better manage excessive and harmful inflammation in children.

## 1. Introduction

Advances in translational medicine and pharmaceuticals over the past three decades have provided pediatric rheumatologists with unprecedented tools to achieve treatment remission without relying on high doses of steroids. Personalized treatment has become common practice, with a focus on targeting specific inflammatory mediators through biological response modifiers. Within this context, interleukin-1 (IL-1) is recognized as a key proinflammatory cytokine involved in the pathogenesis of many autoimmune, autoinflammatory, and immune dysregulatory diseases by initiating a broad range of downstream cascade events [1]. While IL-1α is constitutively expressed by various somatic and hematopoietic cells, IL-1β is primarily produced by myeloid cells upon stimulation.

For everyday practicing providers, there are two commercially available biologic response modifiers designed to antagonize IL-1: anakinra and canakinumab [1,2]. Anakinra is a recombinant IL-1 receptor antagonist (IL-1 RA) that competitively inhibits the binding of IL-1α and IL-1β to their receptor. Canakinumab is a recombinant monoclonal antibody against IL-1β, preventing receptor–ligand interaction. Anakinra is FDA-approved for the treatment of severe rheumatoid arthritis in adults, as well as Neonatal Onset Multisystem Inflammatory Disease (NOMID) and Deficiency of IL-1 Receptor Antagonist (DIRA). Canakinumab is FDA-approved for adult and childhood Still’s disease, Cryopyrin-Associated Periodic Fever Syndromes (CAPS, including NOMID), Tumor Necrosis Factor Receptor-Associated Periodic Syndrome (TRAPS), Hyperimmunoglobulin D Syndrome (HIDS), and Familial Mediterranean Fever (FMF). Targeted treatment against IL-1 is safe and highly effective for these monogenic autoinflammatory conditions in children, as summarized in a recent consensus report by the Pediatric Rheumatology community [3]. The emergency FDA approval of anakinra for the treatment of severe acute respiratory syndrome associated with SARS-CoV-2 further demonstrates its safety, even in the context of controlling infection-triggered inflammation [4].

Anakinra is recognized as an effective steroid-sparing agent with distinct advantages. Its short half-life (<24 h) allows for dose titration without significant concerns regarding long-term adverse effects. Although there has been limited effort to expand its indications for FDA approval, its off-label use for various inflammatory and rheumatological diseases has shown promising results. The literature, to date, includes studies on the safety and efficacy of anakinra in several hundred patients, treating a range of conditions, from Multisystem Inflammatory Syndrome in Children (MIS-C) and Kawasaki Disease (KD) to polyarticular Juvenile Idiopathic Arthritis (JIA), as summarized by Maniscalco et al. [5] and Tegtmeyer et al. [6].

We present our experience with anti-IL-1 agents and their use in treating a range of rheumatological conditions in children, based on current patterns of practice.

## 2. Methods

This study is a single-center, single-provider, real-world evidence analysis conducted through a retrospective chart review of patients managed by the Division of Pediatric Rheumatology at Walter Reed National Military Medical Center (WRNMMC) in Bethesda. Our clinic serves TRICARE beneficiaries aged 0 to 23 years, who are referred as outpatients or through inpatient consultations for autoimmune, autoinflammatory, or immune-dysregulatory conditions within the National Capital Region, as well as from remote U.S. military treatment facilities globally. Access to medications required case-by-case approval by the pharmacy committee. As part of our standard practice, families were actively involved in the decision-making process for off-label treatment regimens, following discussions on the associated risks and benefits.

## 3. Results

### 3.1. Real-World Experience with Anti-IL1 Biologic Response Modifiers

Over the last decade, we have treated 65 patients with anti-IL-1 biologic response modifiers. Our patient population did not include any children with NOMID or DIRA. Anakinra was administered to 63 patients, primarily as an off-label first-line biologic response modifier, typically early in the disease course unless otherwise indicated. Canakinumab was used exclusively for FDA-approved indications in a total of nine patients, as detailed below.

### 3.2. Considerations Prior to Start of Anti-IL-1 Medications

In general, the factors considered prior to initiating anakinra included the likelihood of IL-1 mediated inflammation, the child’s age, the severity of symptoms, the impact of the disease on growth and development, access to care in case of infection, previous treatment regimens, and medication compliance. All patients underwent tuberculosis screening through blood (Quantiferon) or skin (PPD) testing. Baseline laboratory evaluations often included a complete blood count (CBC), liver and kidney function tests, quantitative immunoglobulins (IgG, IgM, IgA, IgE), inflammation markers such as Erythrocyte Sedimentation Rate (ESR), C-Reactive Protein (CRP), and ferritin. If indicated, specific IgG titers (e.g., pneumococcal), T/B/NK cell flow cytometry, CH50 total complement, metabolic status (iron and vitamin D), and potential chronic infections (e.g., EBV, CMV) were also assessed. Additionally, genetic studies were performed using Next Generation Sequencing at Keesler Air Force Base laboratories to screen over 340 genes for mutations causing immune deficiency, immune dysregulation, and inflammation. While this genetic panel is comprehensive, it does not cover all genes involved in autoinflammation, such as TNFAIP3.

### 3.3. Rationale for Off-Label Applications of Anakinra

Based on our experience, the rationale for off-label usage of anakinra can be categorized into four main groups:

1. Treatment of New Patients with Signs and Symptoms of High Systemic Inflammation Undergoing Diagnostic Exclusion:
  This is a critical indication, as no single test can definitively diagnose rheumatologic illnesses, and reaching a conclusion often requires time and close observation. During the initial evaluation, the use of steroids is not advisable while the work-up to rule out infection or malignancy is ongoing. In such cases, anakinra can be employed as a temporary measure to reduce inflammation, especially when a delay in treatment could lead to poor outcomes.
2. Management of Established Patients with Unexpected Escalation of Inflammation Possibly Due to Underlying Infection:
  Rheumatology patients, often on immunosuppressive medications, are particularly vulnerable to infections. When a child presents with fever and elevated inflammatory markers (such as CRP and ESR), distinguishing between a disease flare and an overlapping infection can be challenging. There should be a low threshold for initiating antimicrobial therapy, especially if the patient appears toxic or shows signs of sepsis. While awaiting culture results, if controlling inflammation becomes critical, therapeutic options are limited. In such cases, IL-1RA may serve as a relatively safe alternative to steroids for managing inflammation.
3. The drug can be instrumental in rapidly downregulating endothelial activation and vasculopathy. This is particularly important and challenging when steroid intervention is a concern due to potential adverse effects, such as hypertension, hyperglycemia, hypercoagulation, or in cases involving very young patients.
4. To utilize anakinra as a “litmus test” or pathway discovery tool to gain insights into immunopathogenesis and explore inflammatory pathways is particularly valuable for patients with poorly defined or incompletely understood rheumatological illnesses, especially when the existing literature is insufficient to design an optimal therapeutic regimen. In such cases, inflammation can be managed in a stepwise manner based on the patient’s response to treatment. Typically, treatment with anakinra is limited to short durations (1–4 weeks). For some patients with an established diagnosis, intermittent use of anakinra on an as-needed basis has been beneficial, as discussed below. If long-term anti-IL-1 treatment is necessary, anakinra is often substituted with canakinumab.

### 3.4. Our Experience in Response to Anakinra and Emerging Treatment Protocols

As a consequence of the rationale outlined above, the application of anakinra was found to be safe under the clinical conditions presented in Table 1. For each indication, a reference property (i.e., the treatment target) was instrumental in the overall assessment of treatment response. Close monitoring of both clinical and laboratory parameters was essential to evaluate efficacy levels. In certain cases, assessment was limited to clinical evaluation alone (e.g., the progression of fever, the extent of rash, or synovitis) due to the limited availability of laboratory investigations. Overall, routine blood tests to assess inflammation were limited to ESR, CRP, and ferritin. The CBC was a key test, as it can reflect disease activity—such as leukocytosis in children with sJIA, KD, MIS-C, and some PFS, or leukopenia in cases of MAS or lupus.

Emerging treatment protocols are summarized in Table 2. The patterns reflect the results of experience gained from personalized dosing, which ranges from 1 mg/kg/day to 20 mg/kg/day, and from a few days to extended durations of treatment.

1. *Fever and systemic inflammation:*

Anakinra was the first-line treatment for patients with newly diagnosed systemic-onset Juvenile Idiopathic Arthritis (sJIA or Still’s disease) and macrophage activation syndrome (MAS). It was also used as an adjunct treatment in children with Kawasaki Disease (KD) upon a partial response to intravenous immunoglobulin-G (IVIG) infusion, or if the child presented with coronary artery involvement. Additionally, Anakinra was included in the initial treatment regimen for Multisystem Inflammatory Syndrome in Children (MIS-C) and Acute Rheumatic Fever (ARF), with or without carditis.

Anakinra was also employed as a second-line treatment for selected autoinflammatory diseases, such as periodic fever syndromes (PFS), following a partial response to NSAIDs or colchicine. The immunopathogenesis of these conditions involves a common determinant: the activation of the IL-1 pathway.

  (a) Autoinflammatory and immune dysregulatory diseases:
    A total of 12 patients (9 females) with a mean age of 7.4 years (median age of 7.6 years, ranging from 5 weeks to 20.3 years) with autoinflammatory conditions were treated with anakinra. The indications included FMF (*n* = 4), HIDS (*n* = 3), TRAPS (*n* = 2), PFAPA (*n* = 1), and ill-defined autoinflammatory syndrome (*n* = 3). All FMF patients had single or compound heterozygous mutations in the MEFV gene. Patients with TRAPS and HIDS had genetically confirmed diagnoses.
    Out of the 12 patients, anakinra was initiated as the first-line treatment in two cases: a teenage female with FMF who also had Klippel–Feil syndrome and type 1 diabetes mellitus, and a 5-week-old female who required an intensive care stay upon initial presentation with possible infection versus Sweet syndrome, later found to have HIDS. The remaining patients started on anakinra after a failed response to colchicine. The usual colchicine dose was 0.3 mg/day for toddlers and 0.6 mg/day for older children, titrated up two-fold during flares. When this was inadequate to prevent flares, once-daily injections of anakinra were initiated at an initial dose of 1–3 mg/kg/day, titrated up to 5 mg/kg/day based on treatment response. Patients were then monitored on anakinra PRN during flares on a background of colchicine before considering a transition to canakinumab.
    Of the four patients with FMF, the aforementioned teenager with complex health concerns required long-term twice-daily treatment with 100 mg per dose to control her flares, which also helped normalize her blood glucose levels. A 5-year-old female later transitioned to canakinumab at age 9 with improved disease control. A 6-year-old male remained on PRN anakinra at 100 mg once daily for three consecutive days on a background of colchicine. A 19-year-old female manifesting with flares of low-grade fever, rash, capillary leak, and severe abdominal pain initially benefited from IL-1RA along with IVIG; she was later transitioned to adalimumab seven years later upon establishing a clinical diagnosis of Yao syndrome.
    The patients with HIDS also responded well to anakinra but all transitioned to canakinumab due to dependency on anti-IL-1 treatment and the need for long-term management. The timing of the transition to canakinumab was influenced by the development of injection site reactions and/or the impact of prolonged daily injections on quality of life. Two male siblings, aged 1.3 and 2.6 years, with TRAPS, also progressed to canakinumab within a year of diagnosis after multiple interruptions due to minor respiratory tract infections. The patients with PFAPA and ill-defined autoinflammatory syndromes also benefited from PRN applications of anakinra during flares, administered once daily for 1 to 3 days upon early signs of an upcoming flare, with or without continuing background colchicine treatment. The patients experienced a reduction in the number of flares within six months of starting the drug.
    Two patients received canakinumab as the first-line biologic: a 4-year-old female with FMF, started on canakinumab for convenience due to parental military deployment, and a 6-year-old male with an established diagnosis of TRAPS, who continued his canakinumab treatment initiated at a remote medical center as the first-line treatment. All patients receiving canakinumab remained in full remission.
  (b) sJIA/Still’s disease and MAS:
    Six patients (4 females) aged 5 to 22.8 years (mean age 12 years, median age 12.1 years) were diagnosed with systemic onset juvenile idiopathic arthritis/Still’s disease (sJIA) and treated with anakinra as initial therapy. At disease onset, 5 out of 6 patients exhibited emerging signs of macrophage activation syndrome (MAS), indicated by mild increases in LDH, ferritin, and D-dimer levels. Three patients required hospitalization, and three were co-treated with intravenous steroids. Among these 6 patients, a 13.1-year-old female with a pre-existing diagnosis of sickle cell disease (SCD) and pain crises, along with a 7-year-old male, achieved remission after a few months of treatment. Three patients followed a monophasic disease course and transitioned to canakinumab. One patient, a 22.8-year-old female, experienced flares overlapping with a severe drug reaction to isoniazid or rifampin for latent tuberculosis while on anakinra. She achieved remission after transitioning to ruxolitinib for 18 months and remained in remission without any treatment for over a year, four years after her initial diagnosis.
  (c) MIS-C, KD and ARF:
    Since the first description of MIS-C in early 2020, anakinra has been reported to be efficacious in down-regulating systemic inflammation caused by COVID-19 [7]. The doses used were almost always above the upper limit of the manufacturer’s recommended dose of 8 mg/kg/day. We administered doses up to 10 mg/kg, given intravenously twice a day, without observing any adverse effects in patients with MIS-C who had negative SARS-CoV-2 PCR results. Four patients with MIS-C (two females), with a mean age of 9.9 years (range 1.5 to 15 years), were treated with anakinra along with IV methylprednisolone (10–20 mg/kg/day divided every 6–8 h), IVIG (2 g/kg/dose), aspirin (30 mg/kg/day divided every 8 h), and enoxaparin. All patients had cardiovascular involvement with elevated cardiac enzymes; both males (aged 11.5 and 13.0 years) also had coronary artery aneurysms (CAA). The anakinra dose was titrated down to 2 to 5 mg/kg/day after normalization of cardiac enzymes, followed by a slow wean over the next two weeks. The treatment course was monophasic, allowing for discontinuation of treatment within 4 to 8 weeks, as summarized previously [8]. Echocardiogram (ECHO) at one-year follow-up showed either normalization (initial z-score +5.8) or marked improvement (z-score reduced from +6.0 to +2.8) in coronary artery measurements.
    We treated four patients (two females) with Kawasaki disease (KD) or atypical KD at a mean age of 4.2 years (range 1.5 to 10.9 years) using anakinra. This treatment was administered in conjunction with standard background therapy, including intravenous immunoglobulin (IVIG) and high-dose aspirin for up to 10 days. Two patients received anakinra early in the course due to concerns about emerging macrophage activation syndrome (MAS) or the presence of pancarditis. The other two patients had mild coronary artery dilation and continued to spike fevers despite two rounds of IVIG (2 g/kg/dose). All patients fully recovered, with normalized echocardiograms (ECHO) observed at the two-week follow-up.
    Three patients (two females) diagnosed with acute rheumatic fever (ARF) were treated with anakinra after fulfilling the Jones criteria and demonstrating evidence of prior exposure to Group A beta-hemolytic Streptococcal infection (GABHS). Of the three patients, two (approximately 34 months and 16.6 years old) had carditis, while the third patient (approximately 7 years old) presented with migratory arthritis as the major criterion. The adolescent did not have a fever but exhibited severe mitral regurgitation (MR) and aortic insufficiency. The toddler was initially treated with two rounds of intravenous immunoglobulin (IVIG) and aspirin for incomplete Kawasaki disease (KD). Due to a partial response, indicated by the return of fever, Rheumatology initiated anakinra treatment. The other two patients received anakinra as first-line therapy.
    All three patients showed clinical improvement, with laboratory results for erythrocyte sedimentation rate (ESR) and C-reactive protein (CRP) normalizing within 5 to 10 days of treatment. Echocardiogram (ECHO) findings normalized for the toddler, and the adolescent demonstrated significantly improved ECHO results, with MR reduced from severe to moderate. The adolescent was not a suitable candidate for steroid treatment due to the unclear efficacy of steroids on MR according to the literature, and because his weight was in the 95th percentile for his age. He was treated with anakinra at a dose of 100 mg twice daily for three days, followed by once daily for a week. His laboratory results showed ESR decreased from 55 to 20 mm/h (normal < 28), and B-type natriuretic peptide (BNP) decreased from 1163 to 36 ng/mL (normal < 125). All three patients tolerated the tapering off of anakinra over 7 to 14 days and remained stable thereafter.

2. *Post viral arthritis:*

**Figure 1:**
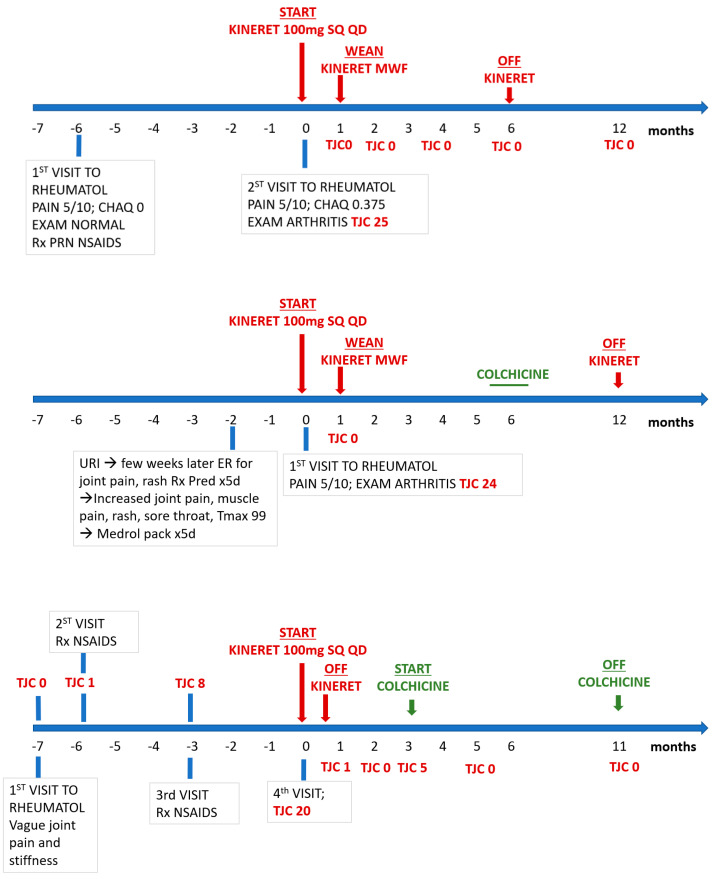
Personalized treatment of representative patients (patients 1, 2, and 3 from top down) for post-EBV arthritis with anakinra with or without colchicine and time course. Readout parameters of disease severity by total joint count (TJC), pain by visual analog scale, from 0 to 10, 10 worse; childhood health assessment questionnaire (CHAQ) from 0 to 3, 3 worse. TJC included joints with varying levels of swelling (including subtle fullness or synovial thickening). Please note differences in patient presentation and time-course on emerging arthritis. Only patient 2 had increased ESR 100 mm/h (normal < 27) and CRP at 6.5 mg/dL (normal < 0.5). EBV IgG titers ranged from low positive (patient 1) to high (patient 3); Patient 3 was also EBV PCR positive and ANA positive. All three patients were Rheumatoid factor negative. Patients 2 and 3 had positive family history of rheumatoid arthritis. NSAIDs, non-steroidal anti-inflammatory drugs MWF, Monday–Wednesday–Friday, SQ, subcutaneously, QD, once daily.

Nine female patients with a mean age of 14.1 years (range 10.1 to 19 years) with new-onset arthritis following recent Epstein–Barr virus (EBV) infection were treated with IL-1 receptor antagonist (IL-1RA) as the first line of immunomodulatory treatment. The diagnosis was based on elevated blood EBV antibody titers; one patient also had a positive EBV PCR. These patients had not improved with NSAIDs prescribed by their primary care providers. The rationale for using anakinra was to address the uncertainty between infectious and post-infectious synovitis, opting for a short-acting targeted treatment before committing to long-term therapy with conventional DMARDs, such as methotrexate or anti-TNF biological response modifiers. Additionally, one patient with a history of a positive Quantiferon test required isoniazid (INH) treatment. All patients presented with fatigue, joint pain, and morning stiffness (ranging from 30 min to all day); two patients also had low-grade fever (<100 °F) along with a transient erythematous non-itchy rash. All patients had a tender joint count (TJC) greater than 14, primarily affecting the hands, wrists, elbows, ankles, and knees. The mean duration between symptom onset and the start of treatment ranged from 1 to 6 months. Figure 1 summarizes the treatment response over time. Similarly, one patient with post-streptococcal chronic reactive arthritis affecting multiple joints responded well to less than one week of treatment with the drug. According to the chart review, this patient transitioned back to Naprosyn as needed and remained well without further need for rheumatology follow-up.

On the other hand, anakinra was marginally effective in two female patients with established diagnoses of juvenile idiopathic arthritis, aged approximately 6 and 12 years, who were partially responsive to anti-TNF treatment. Additionally, a 17.6-year-old male with newly diagnosed rheumatoid factor (RF) positive rheumatoid arthritis did not respond to initial treatment with this drug before transitioning to anti-TNF medication. The latter two patients (the 12-year-old female and the 17.6-year-old male) both tested positive for EBV antibodies.

3. *Mucositis and serositis:*

**Figure 2:**
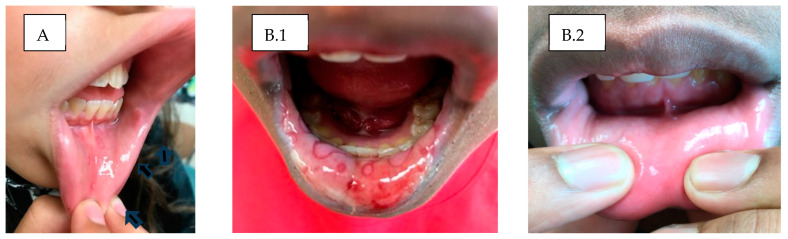
Two patients (**A**,**B**) with painful mouth ulcers. Arrows on patient A point to three small aphthous ulcers that improved on daily colchicine. Patient B with MIRM flare causing severe mucositis affecting oral intake. He was started on anakinra as the first-line medicine for the intensity of mucositis. Pictures before (**B.1**) and after (**B.2**) anakinra 100 mg a day ×3 followed by a brief course of colchicine. Both patients had normal ESR and CRP.

Patients with limited mouth ulcers, such as those seen in PFAPA, were typically first treated with colchicine before considering anakinra. Two patients, aged 12.8 and 17 years, with severe mucositis from incomplete Behcet’s syndrome, which caused recurrent mouth and genital ulcers (without uveitis or systemic concerns such as fever, joint pain, or signs of systemic vasculitis), responded well to intermittent anakinra therapy administered for 3 to 7 days at the onset of new ulcers. Similarly, a 16-year-old male diagnosed with Mycoplasma pneumoniae-Induced Rash and Mucositis (MIRM) responded well to anakinra, which was administered on an as-needed basis for up to 7 days during two flares that occurred 2 years apart (Figure 2(B.1,B.2)). Additionally, two female patients, aged approximately 17 and 23 years, followed in our clinic for subtle recurrent pericarditis that presented with pain and was resistant to colchicine, also responded well to brief, intermittent applications of anakinra for a few daily doses at a time. On this regimen, their flares gradually dissipated within 6 months.

4. *Systemic autoimmune diseases:*

A total of 13 patients (11 females) with a mean age of 14.3 years (median 15.5 years, range 9 to 21.5 years) and systemic connective tissue disease (CTD) were treated with daily anakinra. The cohort included eight patients with systemic lupus erythematosus, three with undifferentiated CTD, and two with Sjogren’s syndrome. The indications for treatment were pericarditis (2 lupus), pleural effusion (1 lupus), polyarticular arthritis (3 lupus, 4 CTD, and 1 Sjogren’s), and macrophage activation syndrome (MAS) (2 lupus). Anakinra was administered at a dose of 100 mg once or twice daily for serositis or MAS during inpatient care (n = 5). The remaining patients received the drug on an outpatient basis at 100 mg once daily for arthritis over 1–2 weeks while continuing background treatment as a steroid-sparing agent. This regimen allowed for clinical stability; however, disease control required further adjustment with other disease-modifying antirheumatic drugs. One patient with Sjogren’s syndrome and chronic leukocytoclastic vasculitis of the lower extremities remained dependent on IL-1RA to maintain clinical remission.

5. *Small vessel vasculitis:*

Two male patients, aged approximately 7 and 10 years, with Henoch–Schoenlein purpura (HSP), and a 13.5-year-old female with alveolar hemorrhage due to ANCA-associated vasculitis (AAV), were treated with daily anakinra. The patients with HSP exhibited target organ involvement of the skin and gastrointestinal tract, presenting with palpable purpura and colicky abdominal pain. Both patients showed a robust response to daily dosing over a one-week treatment period. For the patient with AAV, anakinra was used briefly as an adjunct therapy alongside steroids, immunosuppressives, and B-cell depleting biologic response modifiers; she was able to recover from progressive hypoxia without the need for ventilator support.

## 4. Discussion

Diagnosis and assessment of rheumatological diseases can be particularly challenging, especially when access to subspecialty care is limited. Children may present with life-threatening inflammatory conditions, such as MIS-C, KD, MAS, ARF, vasculitis, and sJIA. Even when inflammation is confined to specific tissues, as seen in cases of serositis, mucositis, or post-viral arthritis, patients often suffer from significant pain and discomfort. Currently, there are only two primary treatment options: non-steroidal anti-inflammatory drugs (NSAIDs) and steroids. NSAIDs, which are weak immunomodulators, are frequently used as antipyretics by inhibiting prostaglandin synthesis. Steroids, while capable of modulating many components of both the innate and adaptive immune systems, can have detrimental adverse effects. Therefore, it is imperative to develop safe and effective treatment options to downregulate inflammation. Based on our real-world observations, anakinra shows promise as a steroid-sparing agent that could effectively address this need.

Rheumatologic diseases can be stratified into three main categories: (1) autoinflammatory diseases, which are monogenic disorders often due to mutations in the IL-1 pathway; (2) acute inflammatory diseases, including reactive conditions such as Kawasaki disease (KD), multisystem inflammatory syndrome in children (MIS-C), acute rheumatic fever (ARF), reactive arthritis, etc.; and (3) autoimmune diseases characterized by the presence of adverse immune memory, such as lupus, myositis, scleroderma, and vasculitis [9]. IL-1 receptor antagonist (IL-1RA) therapy has proven particularly effective for autoinflammatory and acute inflammatory conditions, both of which are primarily driven by innate immunity. IL-1RA can be administered as monotherapy or as part of a combination treatment tailored to the patient. In our experience, short-term treatment is often sufficient to resolve or significantly reduce acute inflammation. In cases of systemic autoimmune diseases, the drug has provided a degree of clinical stabilization, allowing time for proper diagnosis and the development of long-term treatment strategies.

Anti-IL-1 therapy is initiated when the immunopathogenesis of a condition is known or suspected to involve innate immunity. In some cases, a trial-and-error approach may be justified due to the drug’s short half-life, with careful dose titration based on the severity of inflammation, response to treatment, and infection risk. These decisions are often guided by pattern recognition and basic laboratory investigations. Accordingly, we stratified the clinical indications for anti-IL-1 therapy into five categories and the underlying rationale into four domains.

Based on our single-center experience, the following observations on anakinra bear significance: Our review confirms the safety of anakinra when it is used off-label. Based on the guidance by the National Cancer Institute Common Terminology Criteria for Adverse Events (CTCAE) v4.0, anti-IL1 biologic response modifiers were tolerated well. In our hands, there were no serious adverse effects or increase in infection. As reported before [10], injection site reaction was the most common adverse effect for anakinra that required weaning (to every other day) or discontinuation of injections in 15% of cases. There was no allergic reaction to anakinra even at high doses when given through the intravenous route to hospitalized patients. In addition, our experience is also significant as brings the concept of ‘as needed’ applications of anakinra, each for a brief period of time (Table 2). So far, the anti-IL-1biological response modifiers are FDA-approved for the assumption of continuing use. This can be challenging for young patients to endure daily anakinra injections. We used colchicine in combination with the hope of minimizing anakinra use, mostly for the purpose of cost saving. The application was based on the assumption that colchicine can contribute to dampening the inflammation caused by damage-associated molecular pattern (DAMP) molecules in the microenvironment. Furthermore, our data are significant for observing the robust response to anakinra at levels similar to those in the literature for the treatment of MIS-C [11,12], KD [13], oral ulcers [14], serositis [15] and lupus [16,17]. In our hands, patients with polyarticular JIA showed marginal improvement on anakinra similar to published reports [18]. Previous studies have shown Sjogren’s patients with fatigue did not respond to anakinra [19]. Although Sjogren’s is a lymphocyte-driven autoimmune disease, we did observe improvement of leukocytoclastic vasculitis in a teen with Sjogren’s. This was likely to be associated with the effects of anakinra on endothelial cells as our patients with small vessel vasculitis, including HSP, also improved on anakinra. Lastly and perhaps the most novel aspect of our experience was to introduce new off-label applications of anakinra, including post-viral arthritis, post-streptococcal reactive arthritis, MIRM and ARF.

The latter observation highlights a crucial principle: blocking IL-1 is vital for treating acute post-infectious rheumatological diseases. This is a feasible approach since the activation of monocytes and tissue macrophages is a natural immune response during infection. A clear example is observed in the histopathology of tissue samples from patients who succumbed to SARS-CoV-2 [20]. Although the host factors contributing to the emergence of rheumatological illnesses are still poorly defined, activation of innate immunity and the involvement of IL-1 have been clearly shown as common denominators in the pathogenesis of MIS-C, KD, rheumatic fever, post-EBV, and post-streptococcal reactive arthritis [11,21].

The selection of anti-IL-1 medications may evolve over time with emerging evidence and growing literature. Currently, each brand of commercially available anti-IL-1 biological response modifiers offers different benefits based on their chemical and biological properties. The preference for one over another is determined primarily by factors such as half-life, availability, and cost. For instance, canakinumab is used for patients who benefit from blocking the IL-1 pathway. It is preferred for its prolonged half-life of four weeks, making it suitable for children requiring long-term treatment. However, canakinumab is not preferred for patients with a possible infection or an unclear diagnosis. Despite the cost of a month’s supply of canakinumab being nearly ten times higher than that of anakinra, it is an excellent maintenance treatment for a broader range of FDA-approved indications and requires minimal injections per year. There is no need for laboratory monitoring to rule out adverse effects of anakinra or canakinumab, and neither agent causes the development of neutralizing antibodies. In our experience, the sequential application of anakinra and canakinumab has been the safest and most cost-efficient approach.

Access to biologic response modifiers is challenging due to restrictions imposed by the health insurance industry in compliance with FDA guidance. Generally, the pediatric indications for anakinra are limited to NOMID and DIRA, with fewer than 100 reported cases in the literature [22]. Even for polyarticular arthritis, the formulation is FDA-approved only for individuals over 18 years of age. Off-label use is becoming increasingly difficult, even within the military system, despite the critical need for rapid recovery in children to ensure their parents’ full commitment to military readiness. It is important to emphasize that neither anakinra nor canakinumab is a new drug; both have well-established post-marketing safety profiles. Furthermore, the scientific knowledge accumulated over the past five decades through clinical and preclinical studies underscores the fundamental role of IL-1 in many inflammatory conditions. Based on the consensus within the rheumatology community, “targeted interventions” are necessary for the treatment of growing children, as the adverse effects of steroids are inevitable and of great concern, as summarized in a recent commentary; Overcoming the current obstacles to help these children requires a fundamental transformation within the healthcare system [23]. Therefore, it is crucial to develop a patient-centered and industry-friendly approach to expand FDA-approved indications for inflammatory diseases. Achieving this vision will require raising awareness among primary care providers and fostering grassroots discussions that incorporate parent and patient voices as the backbone of advocacy efforts to improve care for these patients.

## Figures and Tables

**Table 1:** An outline summarizing the diagnoses and rationale for off-label applications of anakinra. The intervention was targeted to resolve central concerns (treatment target) and treatment outcomes were based on clinical assessment and laboratory investigations (Readout). The were 29 patients with fever-inflammation, 13 with post-viral arthritis, 5 with mucositis-serositis, 13 with systemic autoimmune disease and 3 with small vessel vasculitis, totaling 63 who were treated with off-label anakinra. Still’s (i.e., systemic onset Juvenile Idiopathic Arthritis); MAS, macrophage activation syndrome; MIS-C, multisystem inflammatory syndrome of children; CAD, coronary artery disease; CMP, complete metabolic panel; CRP, C-reactive protein; ESR, erythrocyte sedimentation rate; BNP, brain natriuretic peptide; ECHO, echocardiogram.

DIAGNOSIS		RATIONALE FOR ANAKINRA	TREATMENT TARGET	READOUT PARAMETERS ON INFLAMMATION
**Fever - Inflammation**	Autoinflammatory disease	Pathway discovery/Treatment	Fever	CBC, ESR, CRP, ferritin
	Still’s disease	Pathway discovery/Treatment	Fever, rash, arthritis	CBC, ESR, CRP, S100A8/S100A9, IL18
	MAS	Treatment while there is possible infection	Cytopenia, coagulopathy	CBC, CMP, CRP, ferritin, LDH, d-dimer
	MIS-C	Treatment	Myocarditis, CAD	CBC, CMP, CRP, ferritin, LDH, d-dimer, BNP, Troponin
	KD	Treatment	CAD	CBC, CRP, ESR, ECHO
	ARF	Treatment	Arthritis, carditis	CBC, ESR, CRP, ECHO
**Post-viral arthritis**		Treatment	Arthritis	Physical exam, CRP, ESR
**Mucositis-serositis**		Treatment	Aphthous ulcers/pericarditis	Physical exam, EKG, ECHO
**Systemic Autoimmune disease**		Treatment while there is possible infection	Arthritis, serositis	Physical exam, CBC, CRP, ESR, imaging
**Small vessel vasculitis**		Treatment	Vasculopathy	Physical exam

**Table 2:** Emerging protocols for application of anti-IL-1 biological response modifiers. sJIA, systemic onset juvenile idiopathic arthritis (i.e., Still’s), MAS, macrophage activation syndrome; PFS, periodic fever syndrome (autoinflammatory disease); MIS-C, multisystem inflammatory syndrome in children; KD, Kawasaki disease; ARF, acute rheumatic fever; HSP, Henoch–Schoenlein purpura; QOD, every other day.

DIAGNOSIS	TREATMENT PROTOCOLS
**Autoinflammatory disease**	May try colchicine first; start anakinra if poor response or ill patient who needs rapid rescue; after documented response, determine if anakinra can be used as needed or consider transition to canakinumab
**sJIA/Still’s disease**	Anakinra with or without steroids --> may transition to maintenance medicines (canakinumab or tocilizumab) in weeks to months based on the response. High risk patients may need combination treatment
**MAS**	High doses of anakinra often with varying levels of steroids based on the severity of MAS --> rapid escalation of treatment if indicated or may need transition to other targeted treatment(s)
**MIS-C, KD, ARF**	High dose anakinra for about a week (on background treatment for MIS-C and KD) --> slow wean over 1–2 weeks then stop
**Post-viral arthritis**	Start anakinra and titrate dose, document response --> may wean to QOD then stop. May try colchicine while weaning anakinra.
**Mucositis**	May try colchicine first if clinically mild case; start anakinra if poor response to colchicine or if patient needs rapid rescue; titrate dose to full recovery; upon documented response, may use anakinra on as needed basis
**Serositis**	May try colchicine first if the case is clinically mild and stable; start anakinra if poor response to colchicine or if patient needs rapid rescue; patient may need steroid combination; titrate dose to full recovery; upon documented response, may use anakinra on as needed basis for isolated recurrent pericarditis
**Systemic autoimmune disease**	Use anakinra on selected patients with fever, arthritis or serositis as a rescue agent for brief periods usually at disease onset or when there is potential for infection. Patient often remains on background treatment
**Small vessel vasculitis**	Start anakinra and titrate dose, document response --> may wean to within 1–2 week(s) to QOD then stop (HSP) or may require longer treatment (connective tissue disease - autoimmune vasculitis).

## Data Availability

The data presented in this study are available on request from the corresponding author due to institutional limitations and regulations.
